# The MOLES System for Planning Management of Melanocytic Choroidal Tumors: Is It Safe?

**DOI:** 10.3390/cancers12051311

**Published:** 2020-05-21

**Authors:** Kelsey A. Roelofs, Roderick O’Day, Lamis Al Harby, Amit K. Arora, Victoria M.L. Cohen, Mandeep S. Sagoo, Bertil Damato

**Affiliations:** 1 Ocular Oncology Service, Moorfields Eye Hospital, London EC1V 2PD, UK; roelofs@ualberta.ca (K.A.R.); lamis.alharby1@nhs.net (L.A.H.); amit.arora@nhs.net (A.K.A.); victoria.cohen@nhs.net (V.M.L.C.); mandeep.sagoo1@nhs.net (M.S.S.); 2 Ocular Oncology Clinic, Royal Victorian Eye and Ear Hospital, Melbourne VIC 3002, Australia; 3 NIHR Biomedical Research Centre for Ophthalmology, University College London Institute of Ophthalmology, London EC1V 9EL, UK; 4 Nuffield Laboratory of Ophthalmology, University of Oxford, John Radcliffe Hospital, Oxford OX3 9DU, UK

**Keywords:** choroidal nevi, choroidal melanoma, scoring system

## Abstract

Purpose: To evaluate the MOLES system for identifying malignancy in melanocytic choroidal tumors in patients treated for choroidal melanoma. Methods: Records of 615 patients treated for choroidal melanoma between January 2017 and December 2019 were reviewed. Patients were excluded if iris and/or ciliary body involvement (106 patients), inadequate fundus photography (26 patients), no images available for review (21 patients) and/or treatment was not primary (11 patients). Demographic data and AJCC TNM Stage were collected. Color fundus and autofluorescence photographs (FAF), optical coherence tomography (OCT) and B-scan ultrasounds were prospectively reviewed. MOLES scores were assigned according to five criteria: mushroom shape, orange pigment, large size, enlarging tumor and subretinal fluid. Results: A total of 451 patients (mean age, 63.9 ± 13.9 years) were included. At treatment, mean largest basal tumor diameter (LBD) and thickness were 10.3 ± 2.8 mm (range, 3.0–23.0) and 4.3 mm (range, 1.0–17.0). All but one (0.2%) had MOLES scores of ≥3. Eighty-two patients were treated after surveillance lasting a mean of 1.5 years. Initially, most (63/82; 76.8%) had a MOLES score ≥ 3. Importantly, none of the 451 tumors had a score of <2, and as such, the MOLES protocol would have indicated referral to an ocular oncologist for 100% of patients. Conclusion: The MOLES scoring system is a sensitive (99.8%) tool for indicating malignancy in melanocytic choroidal tumors (MOLES ≥ 3). If the examining practitioner can recognize the five features suggestive of malignancy, MOLES is a safe tool to optimize referral of melanocytic choroidal tumors for specialist care.

## 1. Introduction

There is scope for improvement in the management of patients with melanocytic choroidal tumors. As choroidal melanomas are infrequently seen in non-subspecialty clinics, up to one third of patients referred to an ocular oncology center for uveal melanoma are found to have a simulating lesion, most commonly a choroidal nevus [1,2], which often has minimal risk of malignancy. At the same time, patients with melanoma frequently experience long delays in referral and diagnosis because their tumor is incorrectly classified as a ‘suspicious nevus’, in many instances because the referring clinician is falsely reassured by its relatively small size. As a result, some patients suffer greater ocular morbidity, visual loss, and perhaps and increased risk of metastasis that may have been prevented by timely referral and treatment. 

Historically, enlargement of a choroidal melanocytic lesion has been regarded as the most reliable indicator of malignancy [3,4] and as such, several risk factors for growth have been reported. The acronym ‘DOCTOR GASS’, was proposed by Harbour [5] based on risk factors identified in the 1970s by Gass [6]. Several of these risk factors were confirmed by Shields et al., who devised a well-known mnemonic ‘To Find Small Ocular Melanoma’ [7,8] later adding ‘Using Helpful Hints Daily.’ [9,10]. As recent years have seen advances in multi-modal imaging techniques, this mnemonic has been revised to ‘To Find Small Ocular Melanoma Doing IMaging,’ with the ‘M’ representing ‘Melanoma hollow’ on ultrasound and ‘DIM’ representing ‘diameter > 5 mm’ on fundus photography [11].

The MOLES program has recently been developed by the senior author (BD) to help non-specialists improve the care they provide to patients with melanocytic choroidal tumors. This comprises: the MOLES acronym, to highlight the clinical signs of choroidal melanoma; the MOLES score for estimating the likelihood of malignancy in melanocytic choroidal tumors; and the MOLES referral guidelines for managing patients according to the tentative diagnosis. MOLES is not indented to be used by ocular oncologists as a tool to select patients for treatment. Unlike the aforementioned scoring systems, the MOLES program aims to empower non-specialists to optimize monitoring and referral decisions without requiring ultrasonography and other imaging techniques, which are not widely available in the community.

The MOLES acronym stands for: Mushroom shape, Orange pigment, Large size, Enlarging tumor and Subretinal fluid. Each of these features is given a score of 0, 1 or 2 according to whether it is absent, borderline, or present. These five features were selected with the aim of making MOLES a highly sensitive, safe scoring system. Mushroom shape is almost pathognomonic for choroidal melanoma. It is included in the MOLES scoring system to ensure that even in the absence of other features, patients with mushroom shaped choroidal lesions will be appropriately referred. Although overlying lipofuscin can be seen in a variety of choroidal pathologies, numerous studies have found orange pigment to be an important risk factor for melanocytic lesion [4,5,7,8,10,11]. The mean largest basal diameter of choroidal nevi documented in the Blue Mountain Eye Study was only 1.25 mm [12], only 13% of nevi are > 5.5 mm [13] and thickness > 2 mm is associated with a significant risk of future growth [8,9,11]. Therefore, large size is included as the third feature in an effort to ensure that the MOLES score reliably identifies otherwise bland lesions that are suspicious primarily because their size. Fourthly, although choroidal nevi may enlarge slowly over a long period of time (0.06 mm/year) [14], a more rapid rate of growth (mean 1.0 mm/year, range 0.0–8.0 mm/year) [9] is indicative of malignant transformation. Finally, the presence of sub-retinal fluid is included as the fifth feature as it has also previously been well documented as a risk factor for growth [4,5,7,9,10,11]. A multivariable analysis of 2355 cases by Shields et al. confirmed the predictive value of orange pigment (*p* = 0.0004), tumor thickness (*p* < 0.0001), basal tumor diameter (*p* = 0.0275), and subretinal fluid (*p*< 0.0001) [11].

The MOLES scoring system categorizes tumors as ‘common nevus’, ‘low-risk-nevus’, ‘high-risk nevus’ and ‘probable melanoma’ according to whether the sum total of these five scores is 0, 1, 2 or >2 respectively. The MOLES protocol advises patients with common nevi to undergo review by a community optometrist every two years, ideally with sequential color photography. For the remaining patients, multimodal imaging assessed by an ophthalmologist is recommended, with referral for such care considered non-urgent for patients with low-risk or high-risk nevi and urgent for patients with probable melanoma. Studies at Oxford Eye Hospital and the Ocular Oncology Service at Moorfields Eye Hospital have shown that a significant proportion of patients referred to these centers have common or low-risk nevi. If managed entirely in the community, these patients, many of whom are elderly, would be spared the cost and inconvenience of having to travel to hospital eye clinics, which may be far from their home. The transfer of care to community optometrists would lighten the burden on hospital clinics, reducing waiting lists and freeing up resources for patients in greater need of urgent specialist care.

While the presence of various combinations of the risk factors for future growth are the basis for counselling and treatment decisions [15], their role in determining the urgency of referrals to an ocular oncologist has not been studied. There will be fears that the MOLES scoring system will delay the treatment of some patients with choroidal melanoma, because the likelihood of malignancy is underestimated. To address these concerns, we performed this study to determine how many patients treated for choroidal melanoma at our center had a MOLES score indicating common or low-risk nevus at the time of treatment. In a subset of patients who were treated after a period of monitoring, we also scored the tumors according to the findings at the first assessment. 

## 2. Methods

We reviewed the electronic medical records of all 615 patients undergoing treatment for uveal melanoma between 1 January 2016 and 31 December 2019 with laser (photodynamic therapy or trans-pupillary thermotherapy), plaque brachytherapy, proton beam radiotherapy or enucleation. The following data were collected: dates of birth, first assessment and treatment; sex; affected eye; therapeutic modality; and presence and size of any extraocular tumor extension. Tumors were staged according to the 8th edition of the American Joint Cancer Committee (AJCC) TNM (tumor, node, metastasis) classification [16]. Patients were excluded if: (i) they had undergone previous treatment for uveal melanoma (11 patients), (ii) the tumor extended anterior to ora serrata, to involve the iris and/or ciliary body (106 patients), and/or (iii) imaging of the tumor was lacking or inadequate (47 patients).

Imaging of the tumor comprised wide-field, color and autofluorescence photography (Optos California (Optos plc, Dunfermline, Scotland)), optical coherence tomography (OCT) (Heidelberg Spectralis; Heidelberg Engineering GmbH, Heidelberg, Germany), and B-scan ultrasonography (ACUSON S2000; Siemens Healthcare Limited, UK). Mushroom shape, orange pigment, large size, enlarging tumor and subretinal fluid were scored and tumors categorized as common, low-risk, high-risk nevus or probable melanoma, as described in Table 1.

Statistical analysis was performed using commercially available software (Stata Statisical Software. StataCorp LP). Variables were assessed for normality with the Kolmogorov–Smirnov and Shapiro–Wilk test. Data are presented as mean ± standard deviation (SD) when normally distributed, and as median, interquartile range (IQR) when not. This study was approved by the Moorfields Eye Hospital clinical audit department (No; 452) and was conducted in accordance with the declaration of Helsinki.

## 3. Results

The cohort comprised 451 patients (230 male, 221 female) with a mean age of 63.9 ± 13.9 years. The tumor was located in the left eye in 241 (53%) patients and the right eye in 210 (47%). At the time of treatment, the mean largest basal tumor diameter (LBD) was 10.3 ± 2.8 mm (range, 3.0–23.0) and the mean tumor thickness was 4.3 mm (range, 1.0–17.0). The MOLES scores were 0, 1, 2 and ≥3 in 0.0%, 0.0%, 0.2% and 99.8% patients respectively, with most patients (82.5%) with scores of 4, 5 or 6. The mean MOLES score was 5 ± 1.2 and followed a normal distribution. By AJCC classification, 47.5% of tumors were T1a and Stage I. The distribution of AJCC Group and Stage was similar amongst MOLES score categories (Table 2). Plaque brachytherapy was the most common treatment (59.1%). Type of treatment was not associated with MOLES score (Table 2).

In patients who were initially observed, the delay had a median of 1.5 years (range, 0.2–3.9) (Table 3). None of these patients had scores of 0 (i.e., ‘common nevus’) or 1 (‘low-risk nevus’). The initial MOLES score was 2 (‘high-risk nevus’) in 23.2% (19/82), and ≥3 (‘probable melanoma’) in the remainder (76.8%; 63/82). Specifically, an initial MOLES score of 3 was seen in 37.8% (31/82), 4 in 23.2% (19/82) and 5 in 15.9% (13/82). Of the ‘high-risk nevi’ (MOLES = 2; 19 cases), all were treated after showing an increase in the MOLES score. This consisted of growth (E = 1 or 2) in all except for one tumor, which developed traces of orange pigment and subretinal fluid that were not initially present. Most high-risk nevi (63%; 12/19) had an initial MOLES score of 2 because of size alone (L = 2); the remaining seven had a size score of 1, with six also showing traces of subretinal fluid and one showing traces of orange pigment. 

In the entire group of tumors that were not immediately treated, there were only two tumors that did not subsequently show an increase in the MOLES score by the time of treatment. Both of these tumors had an initial MOLES score of 4 and both patients were offered treatment at their first visit, but had declined. Following a delay of approximately five months, both patients had re-considered the situation, agreeing to proceed with treatment. One had a basal diameter and thickness of 9.8 mm and 2.9 mm respectively, with traces of orange pigment and subretinal fluid. The other had a basal diameter and thickness of 6.2 mm and 1.6 mm respectively, with confluent orange pigment and traces of subretinal fluid. One patient with a MOLES score of 2 underwent treatment. This tumor, which was located pre-equatorially, had an LBD of 12.8 and a thickness of 5.1 with internal blood flow detected on doppler ultrasonography. 

## 4. Discussion

### 4.1. Main Findings

The main finding of this study is that none of the 451 patients would have suffered a delay in referral to an ocular oncologist if their tumor had been correctly scored with MOLES and management organized according to our recommendations. This is because all the patients in our cohort had a MOLES score of 2 or more. 

### 4.2. Discussion of Aims

The main aim of this study was to determine whether patients with choroidal melanoma would experience delays in referral and treatment because of a low MOLES score. Our findings suggest that such delays are likely to occur only in exceptional cases because none of the 451 patients in our cohort had a low score at the time of treatment or, in the 82 with deferred treatment, at our initial assessment. 

We did not aim to measure the MOLES specificity by determining how many melanocytic choroidal tumors with a high MOLES score never progress. This would have required a large number of patients to be followed up for many years without treatment. It would have been difficult to achieve a sufficient sample size because treatment is considered to be urgent once malignancy is suspected, not least because of concerns about missing any opportunity for preventing metastatic spread. As such, it is to be expected that all patients included in this study underwent treatment as this was the main inclusion criterion.

Similarly, we did not seek to determine how many melanocytic choroidal tumors with a low MOLES score eventually prove to be malignant (either because of transformation to melanoma or because the tumor was malignant in the first instance but resembling a nevus). Such a study would be logistically difficult because prospective data collection would take many years, especially as most tumors with low scores are monitored in the community; furthermore, retrospective analysis of patients seen in hospital would be compromised by a high rate of loss to follow-up as these patients tend to be discharged from our care. The absence of MOLES 0/1 in this study confirms the high sensitivity of the scoring system. In the experience of the senior author (BD) it is extremely rare for melanocytic choroidal tumors to grow if they have clinical features that would give rise to a MOLES score of 0/1.

### 4.3. MOLES Rationale

MOLES is intended as a guide to the intensity with which patients with melanocytic choroidal tumors should be investigated and monitored and whether these are best undertaken by ophthalmologists with special expertise and imaging equipment. It is important to note that the MOLES scoring system is not intended for the assessment of non-melanocytic lesions and was devised to help non-specialists remember five key signs that distinguish choroidal melanomas from nevi, which are serendipitously called ‘moles’ in lay terms. The MOLES is also not intended to be used as a tool to select patients for treatment.

MOLES is designed is to make it possible for clinicians to assess these signs with ophthalmoscopy and/or color photography alone, without the use of specialized equipment. MOLES therefore excludes internal acoustic reflectivity because few primary eyecare providers readily have access to ultrasonography in the community. We recently validated this method using a dataset that comprised mainly nevi, showing that nevi generally scored < 3 and melanomas scored > 2 (Al Harby et al, unpublished data) [17].

Features such as drusen and halo are also excluded, because these features lack statistical significance in differentiating choroidal nevi from melanomas. [11] Visual symptoms have many causes and when caused by a choroidal melanoma the fovea is usually disturbed by the tumor or subretinal fluid. Although choroidal melanomas commonly cause photopsia, these symptoms can also be caused by retinal traction and can be confused with migraine fortification spectra. 

**Figure 1:**
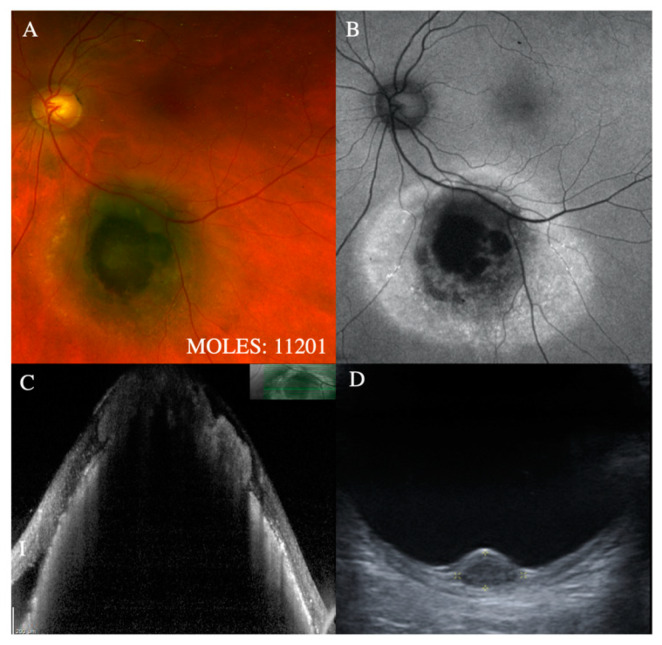
Representative cases demonstrating incipient mushroom shape (i.e., M = 1). (**A**) Fundus photographs showing focal atrophy of RPE, (**B**) highlighted as a well-defined region of hypo-autofluorescence (**C**) with corresponding area of RPE hyperplasia but (**D**) no evidence of a mushroom shape on B-scan ultrasonography (M = 1).

**Figure 2:**
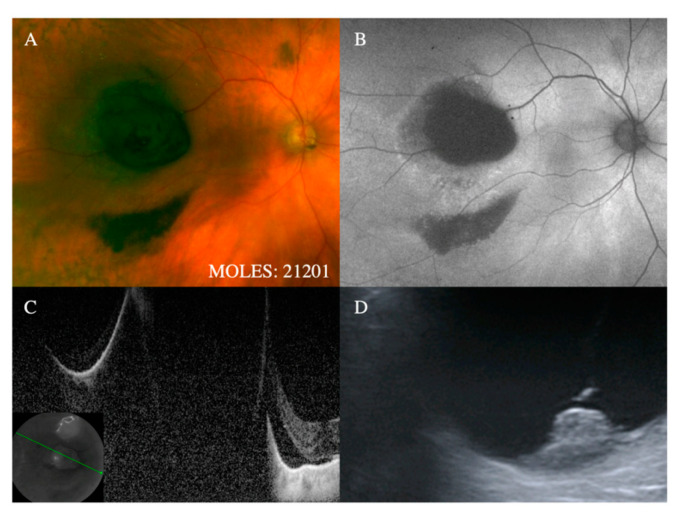
Representative cases demonstrating mushroom shape (i.e., M = 2). (**A**) Evidence of small nodule formation with associated hemorrhage on color photography and corresponding (**B**) hypo-autofluorescence with evidence of (**C**) a nodule on optical coherence tomography (OCT) and (**D**) confirmed on B-scan ultrasound (M = 2).

With regards to the features on which MOLES is based, the mushroom shape is almost pathognomonic for melanoma. Despite this, it is given a score of only 2 to simplify the scoring system on the assumption that tumors with this feature will have other indicators of malignancy, such as increased thickness. Occasionally, choroidal nevi can break through Bruch’s membrane to invade the retina, [18] which is why this feature is given a score of only 1. (Figure 1 and Figure 2).

**Figure 3:**
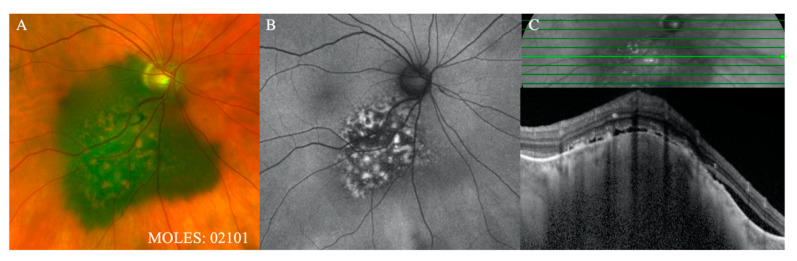
Representative cases demonstrating O = 2. (**A**) Color fundus photograph and (**B**) autofluorescence image demonstrating confluent ‘clumping’ of orange pigment. (**C**) OCT over the tumor confirms the location of lipofuscin superficial to the RPE and also demonstrates the presence of sub-retinal fluid, corresponding to a score of S = 1.

**Figure 4:**
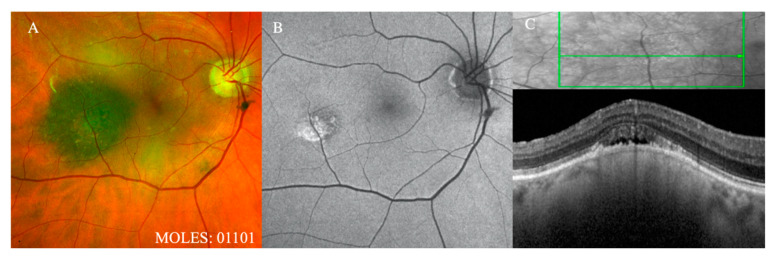
Representative cases demonstrating O = 1. (**A**) Color fundus photograph and (**B**) autofluorescence image demonstrating ‘fine dusting’ of orange pigment. (**C**) On OCT, the lipofuscin is visualized as small hyper-reflective foci lying ‘superficial’ to the RPE, unlike drusen that lie ‘deep’ to the RPE. The presence of trace sub-retinal fluid noted on OCT corresponds to a score of S = 1.

Orange pigment has been shown by several studies to be helpful in differentiating choroidal melanomas from nevi [6,7,8,9,10,11]. Lipofuscin can accumulate over other choroidal tumors, such as metastases and hemangiomas [19,20,21]; however, MOLES is not designed to differentiate between melanocytic tumors and these lesions, which have other diagnostic clues, such as color and shape. Confluent clumps of orange pigment (Figure 3), which should readily be seen with ophthalmoscopy or color photography, are given a score of 2, with a score of 1 reserved for subtle dusting (Figure 4).

With MOLES, tumor thickness > 2mm is given a score of 2 because it is associated with the most significant hazard ratio out of all the risk factors for growth at five years. [11] A study by Augsburger et al. indicates that there are 125 choroidal nevi for every melanoma in the thickness range of 1.5 to 2 mm, 25 nevi for every melanoma in the thickness range of 2 to 2.5 mm, and 5 nevi for every melanoma in the thickness range of 2.5 to 3 mm, approximately [22].

The mean LBD of choroidal nevi documented in the Blue Mountain Eye Study was only 1.25 mm [12]. In a histopathological study of 102 eyes, 13% of nevi were > 5.5 mm [13]. Augsburger et al. found 70 nevi for every choroidal melanoma in the basal diameter range of 5 to 6 mm, 10 nevi for every melanoma in the diameter range of 6 to 7 mm, and 3 nevi for every melanoma in the range 7 to 8 mm, approximately [22]. Without OCT and US, it can be difficult to estimate tumor thickness; however, MOLES minimizes this problem by combining thickness with diameter when categorizing size, because it is rare for melanocytic tumors to grow in thickness without also increasing in diameter. Although choroidal nevi and melanomas overlap significantly with respect to size [22], the discriminatory function of MOLES is enhanced by considering other indicators of malignancy.

Although choroidal nevi may enlarge slowly over a long period of time (0.06 mm/year) [14], a more rapid rate of growth (mean 1.0 mm/year, range 0.0–8.0 mm/year) [9] is indicative of malignancy. A score of E = 1 is assigned when the change in size is minimal or when fundus photography is suggestive of growth but inconclusive due to poor image quality. In this study, there 82 tumors were observed for growth, either because of patients’ preference or due to indeterminate features. Moreover, 63% of those categorized as high-risk nevi received a MOLES score of 2 for size alone, and of course, giant nevi have been previously reported. The policy of observing for growth in these cases was in keeping with conventional practice at the time patients were seen. The present study should enable at least some melanocytic lesions of indeterminate malignancy to be treated without delay.

As with the aforementioned features, the presence of subretinal fluid has also been well documented as a risk factor for malignancy. This feature is given a score of 2 if detectable with ophthalmoscopy and/or color photography and a score of 1 if minimal and detectable only with OCT. If any visual disturbance is found and if this cannot be attributed to unrelated disease, subretinal fluid can be given a score of 1, at the examiner’s discretion, if this seems a plausible explanation for the visual loss. Subretinal fluid overlying nevi with chronic RPE degeneration is usually minimal and should therefore not result in false diagnosis of malignancy because MOLES would require other risk factors to be present. Neovascular membranes over choroidal nevi can cause significant retinal detachment but are rare.

### 4.4. Comparison with other Methods

In the 1970s, Gass identified several features distinguishing choroidal nevi from melanomas [6]. Based on this, Harbour devised the acronym ‘DOCTOR GASS’, which represents: Drusen, Overlying retinal degeneration, Chronic RPE changes, Thickness > 2 mm, Orange pigment, Reflectivity (low) on ultrasonography, Girth (diameter) of tumor, Angiographic hot spots, Subretinal fluid, and Symptoms [5]. Several of these signs were confirmed by Shields et al., who devised a well-known mnemonic ‘To Find Small Ocular Melanoma’, which represented Thickness, Fluid, Symptoms, Orange pigment, and Margin near optic disc [7,8] later adding ‘Using Helpful Hints Daily.’ to represent Ultrasound hollowness, Halo absence and Drusen absence [9,10]. As recent studies showed margin near the disc, absence of drusen and absence of halo to be statistically insignificant, Shields et al. revised this mnemonic to ‘To Find Small Ocular Melanoma Doing IMaging,’ with the ‘M’ representing ‘Melanoma hollow on ultrasound’ and ‘DIM’ representing ‘diameter > 5 mm’ on fundus photography [11] 

Unlike the TFSOM mnemonic and the DOCTOR GASS acronym, MOLES scoring does not require assessment of internal acoustic reflectivity by ultrasonography. Additionally, whereas TFSOM ascribes an all-or-none value to each of its clinical signs, MOLES scores include an intermediate value for features that are borderline, subtle or uncertain. We feel this additional category will be especially useful in the community, where findings are more likely to be inconclusive because of limited experience or equipment. 

### 4.5. Clinical Implications

Singh et al. estimated the overall annual risk of uveal melanoma arising from a pre-existing nevus to be 1 in 8845, with this risk exceeding 1 in 3000 in those older than 70 years [23]. Kivelä et al. took these calculations one step further, taking into account that some uveal melanomas arise de novo, and adjusted the lifetime risk estimate to 1 in 500 [24]. As mentioned, surveillance of common and low-risk melanocytic tumors in the community would reduce costs and inconvenience to patients, most of whom are elderly. Such community care would also reduce hospital waiting lists, allowing limited resources to be allocated to patients with greater need for urgent specialist care.

Currently, a sizable proportion of melanocytic choroidal tumors are categorized as ‘suspicious nevi’, ‘nevomas’ [25] or ‘melanocytic tumors of indeterminate malignancy’ [26]. There is much variation in the management of patients with such lesions. It is hoped that MOLES will make the care of these patients more systematic by splitting these indeterminate tumors into ‘low-risk’ and ‘high-risk’ nevi and defining the clinical features of each of these.

Inevitably, there will be lesions with a low MOLES score that subsequently prove to be malignant. This may occur if clinical signs of malignancy are missed or because a de-novo melanoma is detected at a very early stage, when it may be identical to a common nevus. Such lesions are likely to be small and slow-growing so that serious consequences of mis-diagnosis should be avoided if all patients are reviewed every one to two years in the community. Although not part of the scoring system, visual symptoms should prompt the patient to seek help, initially in the community.

There is a longstanding controversy as to whether or not to treat small choroidal melanomas or whether to observe these until growth is documented [27]. Important and difficult as they are, these dilemmas are not relevant to the MOLES scoring system, which is intended as a guide to recommended investigation and monitoring, not timing and method of treatment. 

The poor correlation between MOLES scores and TNM staging is not surprising given that the two systems of tumor categorization have different purposes, with MOLES indicating likelihood of malignancy and TNM correlating with risk of metastatic death. Similarly, MOLES showed poor correlation with choice of treatment, which is determined not only by tumor dimensions but also factors such tumor distances to optic disc and fovea, which are not relevant to MOLES. 

### 4.6. Implication of the MOLES Scoring System for Tele-Oncology

Tele-oncology platforms for monitoring choroidal and iris nevi have been previously reported [28]. Given that the MOLES scores in this study were purely based on a review of patient imaging, the results are generalizable to tele-ophthalmology platforms. As alternative healthcare delivery platforms continue to be developed, there may be greater scope for remote (‘virtual’) consultation and triaging of patients. Previous studies evaluating optometric referrals found that triage via teleophthalmology reduced office visits to retina specialists by 48%, saving patients cost and time associated with travel and improving efficiency of clinical examination and testing for those requiring treatment [29]. A preliminary assessment of our unpublished data suggests that a similar percentage of referrals for choroidal melanocytic lesions may be avoided by implementing the MOLES scoring system. Innovations in this field have the potential to increase access to sub-speciality care in a cost-effective manner, particularly in geographic areas where the mean number of ophthalmologists falls as low as 9 per million population [30,31]. The COVID-19 epidemic and its anticipated long-term aftermath have increased scope for enhancing these efficiencies [32].

### 4.7. Strengths and Weaknesses of Study

The main strengths of our study are the large number of patients, the multimodal imaging, and the expertise of the ocular oncologists reviewing the images and deciding on patient care. This study has several weaknesses. Some data were missing, because imaging was lacking or inadequate. It was not always possible to estimate the horizontal disc diameter accurately, so that measurements of basal tumor diameter were imprecise. Optical coherence tomography was not possible in many patients, because the tumor was too thick or peripheral. These limitations reflect real world challenges, thereby enhancing the relevance and applicability of our results. 

### 4.8. Implications for Research

Further studies are needed to evaluate the use of the MOLES scoring system in the community. The diagnostic accuracy of this system will depend on the ability of clinicians to recognize the relevant signs of malignancy, with and without the aid of optical coherence tomography, fundus autofluorescence imaging, and ultrasonography. We plan to investigate clinical skills in different groups of practitioners by means of an online quiz, which is in preparation. The success of the MOLES program will depend greatly on educational methods and campaigns that are instituted and these will need to be evaluated. 

There is scope for long-term follow-up studies to determine how many patients ever show growth of their tumor after they are discharged from specialist care for monitoring in the community. This could be done by asking patients or their optometrists to complete a brief electronic questionnaire every one to two years, possibly attaching a color photograph of the lesion to the questionnaire for assessment at the specialist center. 

## 5. Conclusions

Overall, the results of this study support the use of the MOLES scoring system as an aid to optimizing referral of patients with melanocytic choroidal tumors to ocular oncology centers. The timing of MOLES is most opportune in view of the COVID-19 epidemic as it is amenable to being used via tele-oncology platforms, where it may have the potential to increase access to sub-specialty care and early treatment while minimizing unnecessary travel to an ocular oncology center for low-risk lesions. Further study is required to determine the importance of ancillary imaging, such as OCT and fundus and autofluorescence (FAF) on the overall MOLES score and recommended management. Finally, the application of the MOLES scoring system by the targeted end-users, such as general ophthalmologists, optometrists and other primary eyecare providers, is currently underway to evaluate its external validity.

## Figures and Tables

**Table:** **a**.

Risk Factor	Severity	**Score**
**M**ushroom shape	Absent	0
	Unsure/Early growth through RPE	1
	Present	2
**O**range pigment	Absent	0
	Unsure/Trace (i.e., Dusting)	1
	Confluent clumps	2
**L**arge Size	Thickness & Diameter	
	Thickness <1.0 mm (‘flat/minimal thickening’) and diameter < 3DD	0
	Thickness = 1.0–2.0 mm (‘subtle dome shape’) and/or diameter = 3–4 DD	1
	Thickness >2.0 mm (‘significant thickening’) and/or diameter > 4DD	2
**E**nlargement	None (or lesion not documented or mentioned to patient previously)	0
	Unsure (i.e., Poor image quality)	1
	Definite (confirmed with sequential imaging)	2
**S**ubretinal fluid	Absent	0
	Trace (if minimal and detected only with OCT)	1
	Definite (if seen without OCT)	2
	**Total Score**	

DD = disc diameter (=1.5 mm); *ignore thickness if this cannot be measured; **assume SRF if unexplained visual loss.

**Table:** **b**.

MOLES Score	Suggested Management
**0 = Common naevus**	**Monitoring in community** with color photography every 1–2 yrs.
**1 = Low-risk naevus**	**Non-urgent referral** for specialist investigation comprising wide-field photography, autofluorescence imaging, optical coherence tomography and, in selected cases, ultrasonography. Subsequent surveillance to be undertaken at a specialist clinic or in the community according to risk of malignancy.
**2 = High-risk naevus**	
**3 = Probable melanoma**	**Urgent referral** to ophthalmologist with urgent onward referral to ocular oncologist if suspicion of malignancy is confirmed.

**Table 2:** Demographic data, American Joint Cancer Committee (AJCC) stage and treatment stratified by MOLES score for 450*.

Variable	Category	Moles Score						Total	
		3	4	5	6	7	8		
		%	%	%	%	%	%	*N*	%
**Sex**	Female	55.9	50.0	50.6	51.2	61.8	20.0	230	51.1
	Male	44.1	50.0	49.4	48.8	38.2	80.0	220	48.9
**Eye**	L	52.9	49.2	54.8	51.2	64.7	60.0	240	53.3
	R	47.1	50.8	45.2	48.8	35.3	40.0	210	46.7
**Age (Yrs)**	≤55	35.3	27.9	29.8	22.0	5.9	20.0	118	26.2
	55.1–65.0	29.4	23.0	22.0	24.4	29.4	30.0	108	24.0
	65.1–75.0	14.7	32.8	30.4	31.7	47.1	50.0	143	31.8
	>75	20.6	16.4	17.9	22.0	17.6	0.0	81	18.0
**TNM Size Group**	T1	70.6	43.4	48.8	47.6	38.2	50.0	216	48.0
	T2	26.5	36.1	36.9	28.0	55.9	40.0	161	35.8
	T3	2.9	13.9	11.3	22.0	5.9	10.0	58	12.9
	T4	0.0	6.6	3.0	2.4	0.0	0.0	15	3.3
**TNM Prognostic Group**	T1a	69.7	43.0	49.1	45.7	38.2	50.0	212	47.5
	T1c	0.0	0.0	0.0	1.2	0.0	0.0	1	0.2
	T2a	27.3	36.4	35.3	27.2	55.9	40.0	157	35.2
	T2c	0.0	0.0	1.2	0.0	0.0	0.0	2	0.4
	T3a	3.0	12.4	11.4	22.2	5.9	10.0	56	12.6
	T3c	0.0	1.7	0.0	0.0	0.0	0.0	2	0.4
	T4a	0.0	5.0	3.0	2.5	0.0	0.0	13	2.9
	T4e	0.0	1.7	0.0	1.2	0.0	0.0	3	0.7
**TNM Stage**	1	69.7	43.0	49.1	45.7	38.2	50.0	212	47.5
	2	27.3	36.4	35.3	28.4	55.9	40.0	158	35.4
	3	3.0	12.4	11.4	22.2	5.9	10.0	56	12.6
	4	0.0	6.6	4.2	2.5	0.0	0.0	17	3.8
	6	0.0	1.7	0.0	1.2	0.0	0.0	3	0.7
**Treatment**	Laser	5.9	1.6	3.6	1.2	0.0	20.0	13	2.9
	Plaque	76.5	55.7	58.3	58.5	58.8	60.0	266	59.1
	Proton	11.8	24.6	24.4	17.1	23.5	10.0	98	21.8
	Enucleation	5.9	18.0	13.7	23.2	17.6	10.0	73	16.2
**Total**	Number	34	122	168	82	34	10	450	

*one patient with a MOLES score of 2 was excluded from this table.

**Table 3:** Demographic data, AJCC stage and treatment stratified by MOLES score for 82 patients who were observed prior to being treated.

Variable	Category	Moles Score						Total	
		3	4	5	6	7	8		
		%	%	%	%	%	%	N	%
Sex	Female	100.0	33.3	50.0	51.9	60.0	0.0	40	48.8
	Male	0.0	66.7	50.0	48.1	40.0	100.0	42	51.2
Eye	L	100.0	83.3	59.1	33.3	60.0	66.7	44	53.7
	R	0.0	16.7	40.9	66.7	40.0	33.3	38	46.3
Age	<=55	0.0	0.0	36.4	18.5	0.0	16.7	14	17.1
	55.1–65.0	0.0	16.7	18.2	29.6	40.0	16.7	22	26.8
	65.1–75.0	0.0	16.7	22.7	37.0	45.0	66.7	29	35.4
	>75	100.0	66.7	22.7	14.8	15.0	0.0	17	20.7
TNM Size Group	T1	100.0	66.7	72.7	66.7	55.0	83.3	55	67.1
	T2	0.0	33.3	27.3	25.9	45.0	16.7	25	30.5
	T3	0.0	0.0	0.0	3.7	0.0	0.0	1	1.2
	T4	0.0	0.0	0.0	3.7	0.0	0.0	1	1.2
TNM Prognostic Group	T1a	100.0	66.7	76.2	61.5	55.0	83.3	53	66.3
	T1c	0.0	0.0	0.0	3.8	0.0	0.0	1	1.3
	T2a	0.0	33.3	19.0	26.9	45.0	16.7	23	28.8
	T2c	0.0	0.0	4.8	0.0	0.0	0.0	1	1.3
	T3a	0.0	0.0	0.0	3.8	0.0	0.0	1	1.3
	T4a	0.0	0.0	0.0	3.8	0.0	0.0	1	1.3
TNM Stage	1	100.0	66.7	76.2	61.5	55.0	83.3	53	66.3
	2	0.0	33.3	19.0	30.8	45.0	16.7	24	30.0
	3	0.0	0.0	0.0	3.8	0.0	0.0	1	1.3
	4	0.0	0.0	4.8	3.8	0.0	0.0	2	2.5
INITIAL Moles Score	2	100.0	50.0	27.3	25.9	10.0	0.0	19	23.2
	3	0.0	16.7	72.7	29.6	25.0	16.7	31	37.8
	4	0.0	33.3	0.0	40.7	15.0	50.0	19	23.2
	5	0.0	0.0	0.0	3.7	50.0	33.3	13	15.9
Treatment	Laser	0.0	0.0	4.5	0.0	0.0	33.3	3	3.7
	Plaque	100.0	100.0	77.3	85.2	80.0	66.7	67	81.7
	Proton	0.0	0.0	18.2	14.8	20.0	0.0	12	14.6
Total	Number	1	6	22	27	20	6	82	100%
